# Surgical Colonic Myeloid Sarcoma Following Bone Marrow Transplantation in a Patient With Acute Myeloid Leukemia With Myelodysplasia-Related Changes: A Case Report

**DOI:** 10.1016/j.radcr.2026.02.027

**Published:** 2026-03-16

**Authors:** Tamaki Ichikawa, Makiko Kobayashi, Kitaro Irwan Bin Mohd Azlan, Shunro Matsumoto, Shinichiro Machida, Hiroshi Kajiwara, Jun Hashimoto

**Affiliations:** a Department of Radiology, Tokai University School of Medicine, Isehara, Japan; b Department of Radiology, Oita City Medical Association’s Almeida Hospital, Oita, Japan; c Department of Hematology/oncology, Tokai University School of Medicine, Kanagawa, Japan; d Department of Pathology, Tokai University School of Medicine, Tokyo, Japan

**Keywords:** Acute myeloid leukemia, Myeloid sarcoma, Colon

## Abstract

Myeloid sarcoma (MS), a rare extramedullary manifestation of acute myeloid leukemia, can develop in various anatomical sites, including the skin, lymph nodes, and bones. Gastrointestinal involvement is uncommon, and colonic MS is particularly rare. We describe the case of a 60-year-old man with a history of acute myeloid leukemia with myelodysplasia-related changes who underwent bone marrow transplantation. He presented with diarrhea, and abdominal computed tomography revealed bowel obstruction caused by a large ascending colon mass with associated lymphadenopathy. Emergency surgery was performed, and histopathological examination confirmed the diagnosis of colonic MS. One year and 6 months after surgery, leukemic infiltration was detected in multiple organs, including spinal cord, cerebellum, and bone marrow, on positron emission tomography-computed tomography. The patient died about 4 years after the initial presentation. This was the first surgical case of colonic MS caused by bowel obstruction without intestinal perforation. Diagnosis of colonic MS is difficult because of its various radiological findings; however, it may follow as aggressive clinical course, in as our case. This case highlights the radiological and clinical characteristics of surgically managed colonic MS cases in literature.

## Introduction

Myeloid sarcoma (MS), also known as chloroma or granulocytic sarcoma, is a rare extramedullary neoplasm composed of primitive myeloid cells [[1], [2], [3]]. In 2002, the World Health Organization Classification of Myeloid Neoplasms officially listed “myeloid sarcoma” as a subset of acute myeloid leukemia (AML) [1]. Although most seen in patients with AML, MS may also arise during chronic myeloid leukemia, myelodysplastic syndrome (MDS), or myeloproliferative disorders [[1], [2], [3], [4]]. The reported incidence of MS varies widely, ranging from 2.5% to 30% of AML cases [5]. The occurrence of MS is associated with a poor overall survival, with a median survival of 15.9 months [4]. Most frequently affected sites include bone, skin, and lymph nodes, although MS can involve virtually any organ [[1], [2], [3], [4]]. MS of the colon is rare [[4], [5], [6], [7], [8], [9], [10], [11], [12], [13]]; in a series of 83 MS lesions, Meyer et al. [4] reported none in the colon. Eight surgical cases of colonic MS were reported because of intestinal perforation, intestinal pneumatosis, paracolonic abscess, and inflammations [5,[7], [8], [9], [10], [11], [12], [13]]. Radiological features of colonic MS such as polypoid lesions, masses, wall thickening, or ulceration are nonspecific [5,6], and small polypoid lesions have also been detected on colonoscopy [5,6]. We present a surgical case of colonic MS causing bowel obstruction in a patient with a history of AML with myelodysplasia-related changes (MRC) who had undergone bone marrow (BM) transplantation.

This was the first surgical colonic MS case of large mass presenting with bowel obstruction without intestinal perforation. The solid mass of colonic MS could not be distinguished from lymphoma. This case emphasizes the clinical and radiological features of surgical colonic MS.

### Case Report

A 60-year-old male presented with abdominal pain, fever, and diarrhea.

Clinical history: The patient had a history of AML with MRC and had undergone allogeneic hematopoietic stem cell transplantation 3 years ago. The patient remained in complete remission for 1 year and 10 months following BM transplantation.

Laboratory results: Initial blood tests revealed mild leukocytosis (WBC 14.5 × 10^3^/µL), anemia (Hb 12.6 g/dL), and thrombocytopenia (Plt 10.1 × 10^4^/µL). Serum LDH, IL-2R, CEA, and CA19-9 levels were within normal limits; however, WT1 mRNA was elevated (580 copies/µg).

**Fig. 1:**
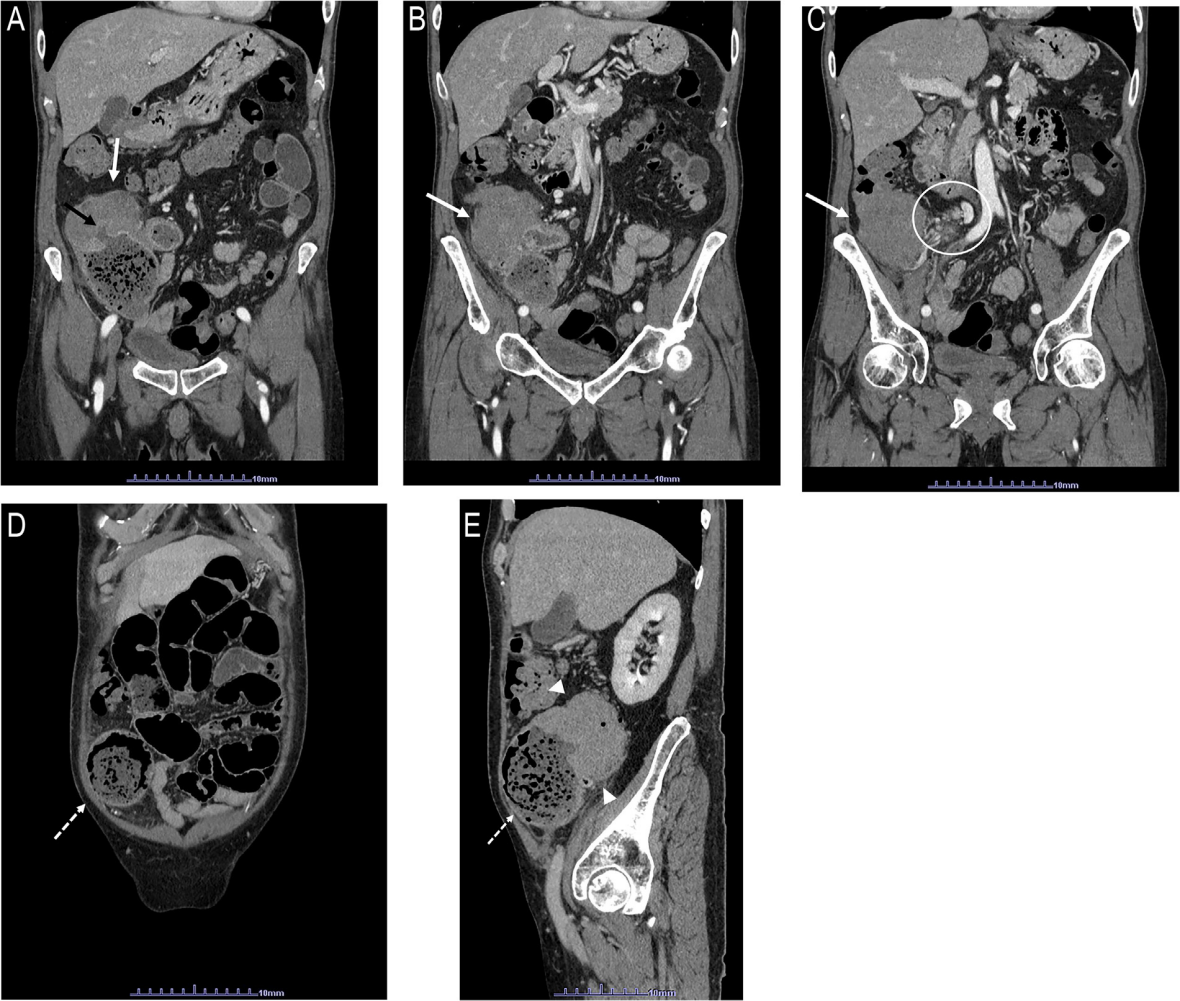
Dynamic computed tomography (CT) images (A–E). Dynamic CT of abdomen was performed 100 mL of isohexol at rate of 4 mL/s. Scanning delay after injection: early phase; 40 s. delayed phase; 120 s. Early coronal images reveal a large solid mass without calcification (white arrows) occupying intestinal lumen of the ascending colon (A–C). Ulceration of the mass (black arrow) is suspected (A). A few enhanced lymph nodes are seen (circle: C). *Note:* Marked dilatation of the cecum (dotted arrows: D, E) and small intestine consistent with bowel obstruction (A, D, E). Delayed sagittal image (E) reveals the solid mass with mild homogenous enhancement (arrowheads).

Imaging findings: Abdominal ultrasonography revealed thickening of the ascending colon wall, prompting dynamic contrast–enhanced computed tomography (CT). CT imaging demonstrated a large, solid non-calcified mass with a maximum diameter of 7 cm in the proximal ascending colon, accompanied by dilation from the cecum to the ileum (Fig. 1A–D). The mass showed mild homogeneous enhancement (75 HU) with ulceration, and associated lymphadenopathy was noted in the delayed phase (Fig. 1C).

**Fig. 2:**
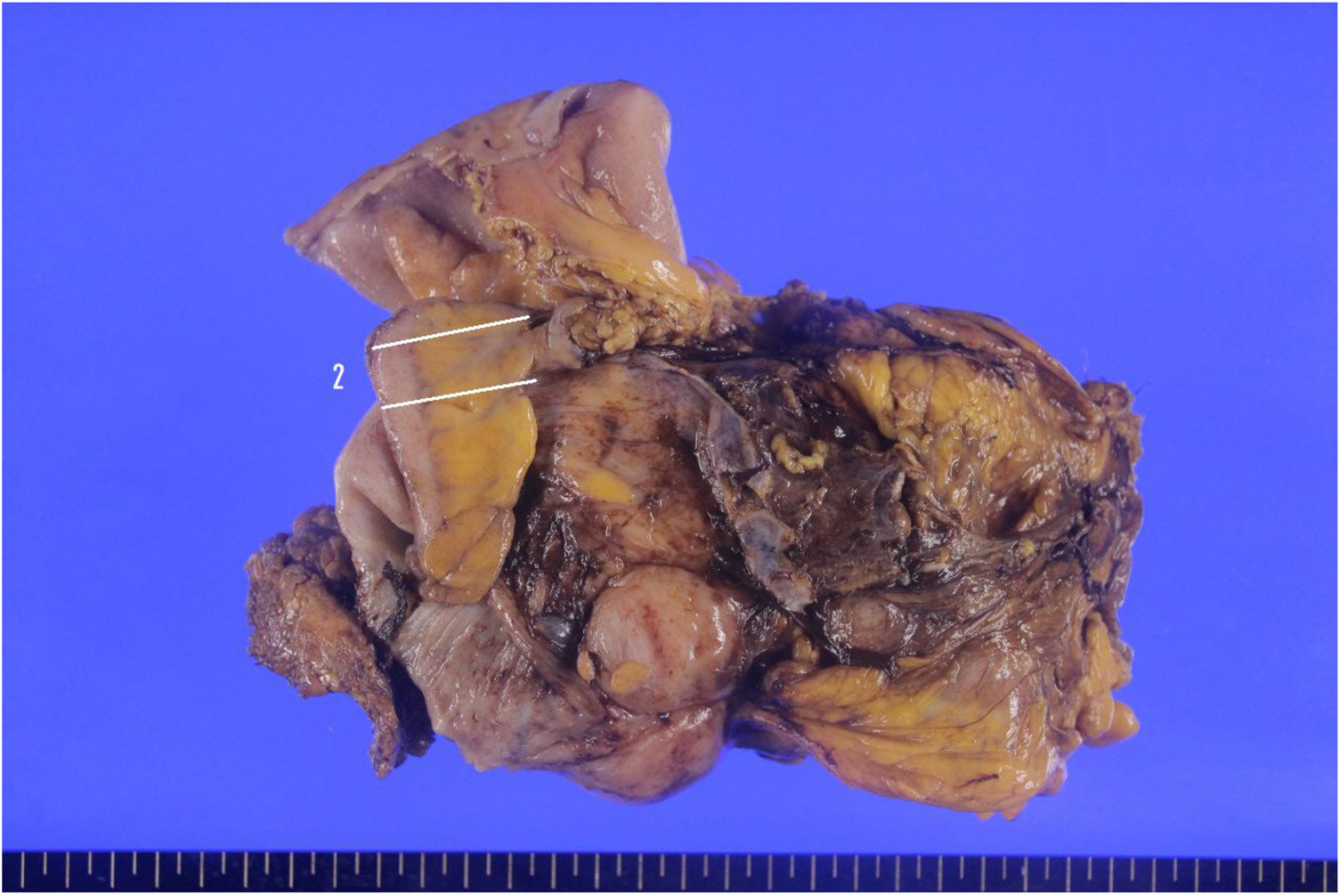
The gross specimen revealed a Borrmann type 3 mass in the ascending colon**.**

Surgical findings: Although no intraperitoneal free air was observed, the patient complained of strong abdominal pain. The emergency surgery was considered due to severe bowel obstruction by colonic mass. Preoperative differential diagnoses included MS and post-transplant lymphoproliferative disorders such as malignant lymphoma. The patient underwent laparoscopic tumor resection of ascending colon with lymphadenectomy. Intraoperatively, a Borrmann type 3 tumor was identified in the ascending colon (Fig. 2).

**Fig. 3:**
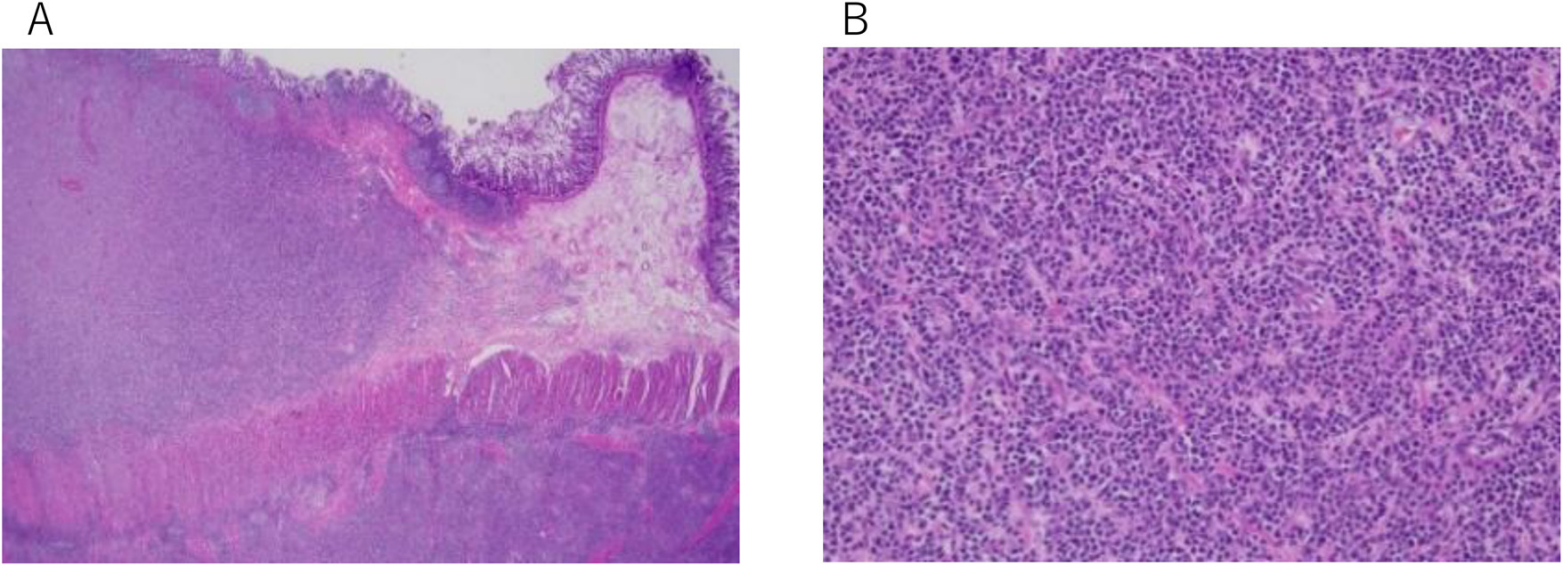
Pathological images of hematoxylin and eosin stains (A: × 2, B: × 200). Hematoxylin and eosin staining demonstrated diffusely infiltrating blasts throughout the intestinal wall.

**Fig. 4:**
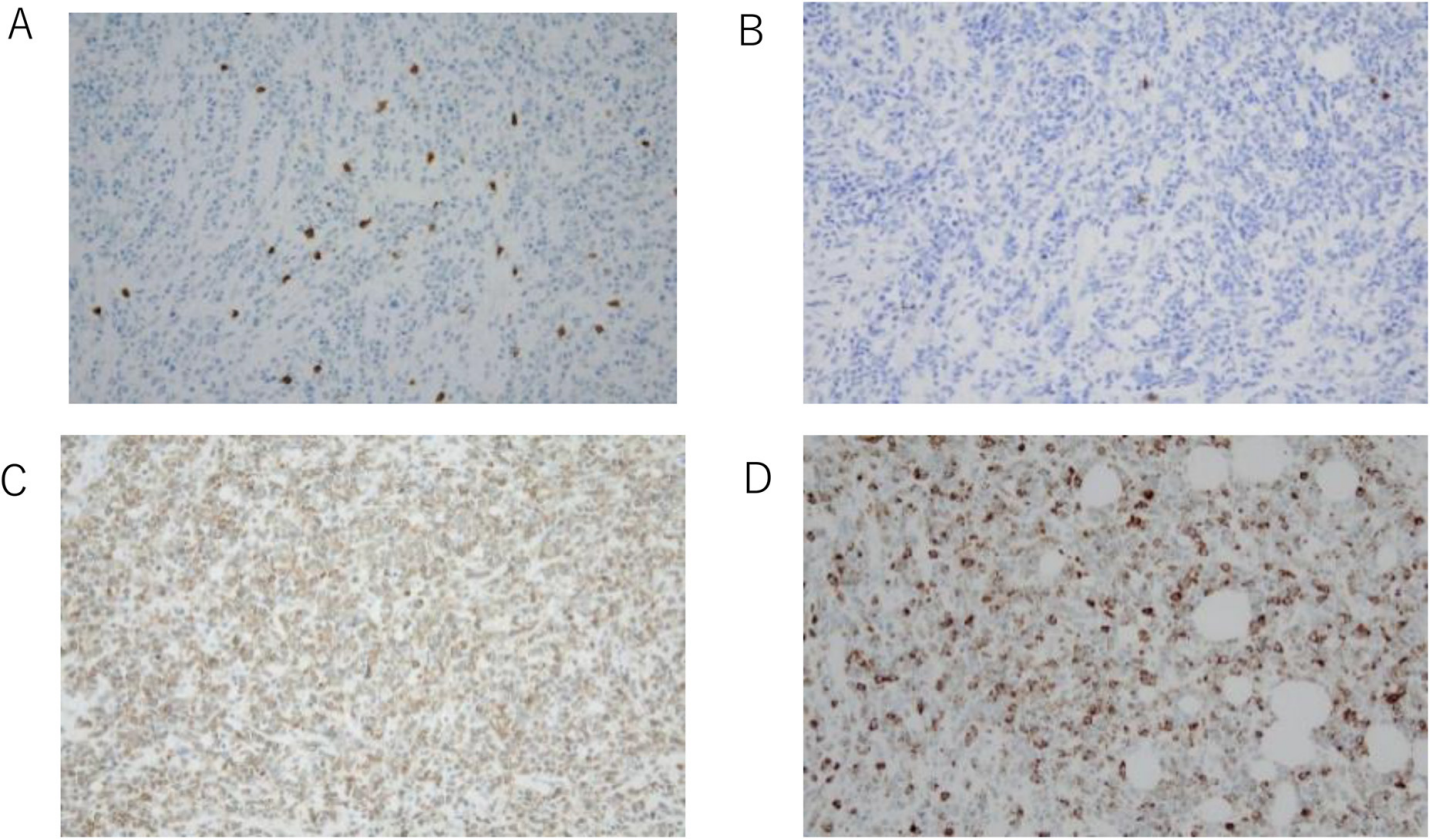
Immunohistochemical images. In the immunohistochemistry, the tumor cells are diffusely positive for CD117 and occasionally positive for MPO. The tumor cells are negative for CD3 and CD20. (A): CD3, (B): CD20, (C): CD117, (D): MPO.

Histopathological findings: Histopathological examination revealed diffuse infiltration of blasts throughout the intestinal wall (Fig. 3). Immunohistochemistry showed that the tumor cells were diffusely positive for CD117 and focally positive for MPO, confirming myeloid lineage, while negative for the lymphoid markers CD3 and CD20 (Fig. 4). Similar abnormal cells were detected in the lymph nodes. The final pathological diagnosis was MS of the ascending colon.

**Fig. 5:**
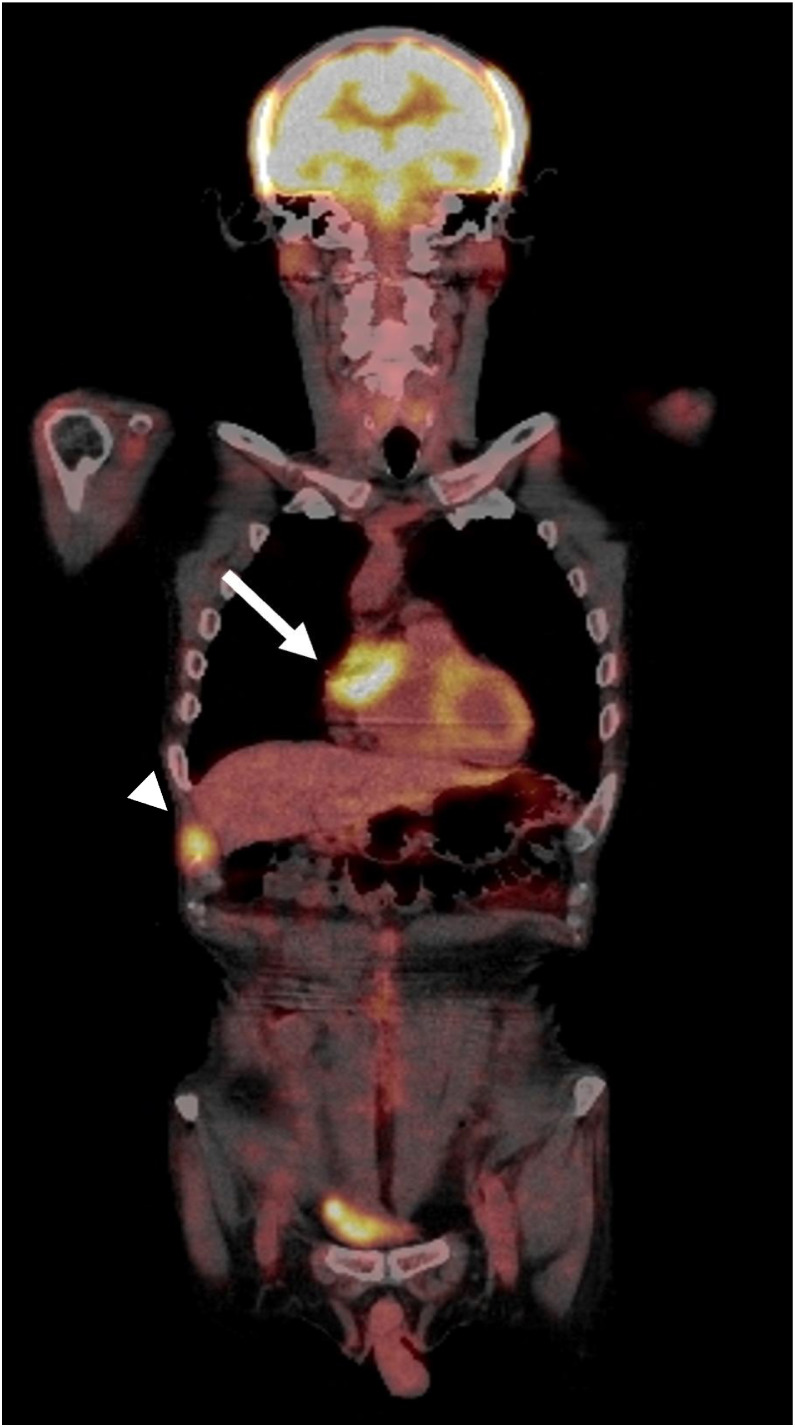
Positron emission tomography (PET)–computed tomography (CT) image 1 year after surgery. PET–CT image reveals 18F-fluorodeoxyglucose (FDG) uptakes in right atrial appendage (arrow, SUV_max_: 7.7) and right chest wall (arrowhead, SUV_max_: 6.3).

Postoperative course: Three months after surgery, positron emission tomography (PET)–CT (Fig. 5) demonstrated 18F-fluorodeoxyglucose (FDG) avid metastases in the right atrial appendage (SUV_max_: 7.7) and right chest wall (SUV_max_: 6.3). The patient received chemotherapy with azacitidine and venetoclax of 11 courses because the chemotherapy was effective before surgery. However, it was ineffective after surgery. His prognosis might be poor because WT1 mRNA levels were continuously rising. Leukemic infiltration was detected in the spinal cord, cauda equina, meninges, brainstem, cerebellum, and BM 1 year and 6 months after surgery. The patient died 3 years and 11 months after his initial presentation.

## Discussion

MS rarely involves the gastrointestinal (GI) tract. When present, the small bowel—particularly the ileum—is the most affected site [6,14]. The reported incidence of GI involvement ranges from 6.5% to 13% [5]. Colonic MS is exceedingly rare, with fewer than 30 adult cases documented in the English literature [[5], [6], [7], [8], [9], [10], [11], [12], [13],^15^,^16^]. Clinical manifestations of MS of GI tract are often nonspecific [5,6,14]. The most common clinical findings include abdominal pain, diarrhea, and intestinal bleeding, which are also frequently encountered during the aplastic phases of AML in patients receiving intensive chemotherapy [5,13,16]. CT findings of bowel MS are highly variable, with lesions appearing as intraluminal or exophytic polypoid masses, bowel wall thickening, or a combination of both [5,13,16]. Patterns of contrast enhancement also vary [5,6,14]. Consequently, bowel MS cannot be reliably distinguished on CT from lymphoma, other neoplastic processes, or inflammatory bowel disease [5,6,14]. In colonic MS, wall thickening with a mass effect is the most frequently reported radiologic and endoscopic feature [5]. However, Elasharawi et al. [16] described a case of non–mass-forming colonic MS without significant radiological or endoscopic findings. The most common complications of bowel MS include hemorrhage, perforation, necrosis, obstruction, and intussusception [5,6,14]. Eight surgical cases of colonic MS (Table 1) have been reported in association with intestinal perforation, pneumatosis, pericolonic abscess, or inflammatory changes [5,[7], [8], [9], [10], [11], [12], [13]]. Two cases of colonic MS were operated due to perforation, and one of them was after BM transplantation case. Aslan et al. [14] also described a rare case of ileal MS presenting as small bowel obstruction. In the present case, emergency surgery was required due to severe bowel obstruction caused by a large ascending colon mass, without evidence of perforation. To the best of our knowledge, this is the first reported surgical case of bowel obstruction caused by a large mass-forming MS of the ascending colon without perforation, in a patient with a history of AML with MRC following BM transplantation.

**Table 1:** Clinical and radiological findings of surgical cases of colonic myeloid sarcoma.

Study	Age, sex	Coexisting hematologic malignancy	Clinical manifestation	Radiological/endoscopic findings	Site	Outcome
1 [7]	60 y, M	Leucopenia and thrombocytopenia	Diarrhea and fever	Diffuse parietal thickening	RC	Death due to toxic shock
2 [8]	28 y, F	Previous AML (post-transplantation)	Abdominal pain	Pneumatosis	TC	Death due to pneumonia
3 [9]	57 y, M	Concomitant CMML evolving in AML	Abdominal pain	Thickening of the wall	SC	Death due to DIC
4 [10]	76 y, F	No other diseases	Negative	Negative	RC	Stable status 3 y
5 [11]	63 y, M	Concomitant AML	Abdominal pain and constipation	Thickening of the wall	SC	Unknown
6 [12]	49 y, M	Pre-existing AML	Abdominal pain	Soft tissue mass	LC	Death due to septic shock
7 [13]	76 y, M	Pre-existing MDS	Abdominal pain, nausea vomiting, and diarrhea	Thickening of the wall of RC intraluminal lesion of TC	RC TC	No operative complication
8 [5]	53 y, M		Abdominal pain	Ischemia of colon wall and perforation	DC, SC, and rectum	Death due to sepsis
9: Present case	60 y, M	AML with MRC (post-transplantation)	Abdominal pain, fever, and diarrhea	Solid mass, dilated cecum and ileum (bowel obstruction)	AC	Death due to MS recurrence

AML, acute myeloid leukemia; AS, ascending colon; CCML, chronic myelomonocytic leukemia; DIC, disseminated intravascular coagulation; DS, descending colon; F, female; LC, left colon; M, male; MDS, myeloid dysplastic syndrome; MRC, myelodysplasia-related changes; MS, myeloid sarcoma RC, right colon; SC, sigmoid colon; TC, transverse colon.

18F-FDG PET–CT is a valuable modality for the early detection of MS, identifying disease relapses before morphological changes become apparent on conventional cross-sectional imaging, as well as for assessing residual metabolic activity after treatment [6,12,17]. In our case, post-surgical 18F-FDG PET–CT demonstrated metastatic lesions with high FDG uptake in the right atrial appendage and right chest wall (Fig. 5). Diffusion-weighted imaging may also serve as a useful diagnostic tool for MS, as diffusion restriction with a mean apparent diffusion coefficient value of 0.57 ± 0.18 × 10^− 3^ mm^2^/s [4]. However, it should be noted that restricted diffusion is not specific to MS and can also be observed in other highly cellular tumors or tumor-like lesions, such as lymphomas, poorly differentiated carcinomas, and abscesses [4].

MS presents in 4 clinical forms: (1) concurrent with AML; (2) as a precursor to AML; (3) in association with MDS progressing to leukemia; and (4) as isolated disease [6,18]. It may arise at any stage of AML: several months before BM involvement, simultaneously with BM disease at AML diagnosis, or during relapse, regardless of BM remission status [18]. Diagnosing de novo MS is particularly challenging radiologically, as it is often misinterpreted as lymphoma due to overlapping immunobiological profiles [14,18]. Accurate recognition is clinically critical, as it directly impacts management [6]. In patients without evident hematologic abnormalities at diagnosis who undergo surgery without systemic chemotherapy, acute leukemia develops in 80%-90% of cases within an average of 11 months following MS diagnosis [6]. The most widely recommended treatment for MS is conventional AML-based chemotherapy protocols [12]. However, surgical intervention is warranted in complicated bowel MS presenting with hemorrhage, perforation, obstruction, or intussusception. In our case, colonic MS was suspected due to elevated WT1 mRNA levels, and emergency surgery was necessary to relieve bowel obstruction identified on CT.

Genetic profiling has revealed recurrent genetic abnormalities, including specific mutations associated with MS [19]. Genetic alterations linked to AML-MRC are frequently correlated with unfavorable prognostic features [20]. The genetic abnormality identified was a deletion 7q. Monosomy 7 and 7q deletions are the most frequent chromosomal abnormalities in AML and are associated with adverse outcomes and high relapse rates [21]. However, prognosis of MS patients with 7q deletions is unknown.

Prognosis in MS is influenced by several factors, including the anatomical site involved, timing of diagnosis, underlying genetic profile, and treatment approach [[4], [5], [6],^14^]. Large retrospective studies have not shown a clear prognostic difference between patients with MS and those with AML without MS or those with synchronous AML [22]. However, a prior history of MDS or MPN has consistently been associated with poorer survival outcome [22].

The imaging findings of MS are nonspecific, and differential diagnoses such as inflammatory or other neoplastic lesions must also be considered. However, in patients with AML who have undergone hematopoietic stem cell transplantation, the presence of a large colonic mass should raise suspicion for colonic MS. When CT imaging reveals bowel obstruction caused by a colonic mass, surgical intervention should be considered proactively. Buzzati et al. [5] reported 2 cases of colonic MS associated with adenocarcinoma among 7 surgical cases of MS. Therefore, biopsy or surgical resection is essential to establish a definitive diagnosis of suspected colonic MS. Radiologists should keep in mind developing colonic MS in even AML patients in complete remission for imaging surveillance.

## Conclusion

We present an emergency surgical case of bowel obstruction caused by colonic MS in a patient with a history of AML with MRC who had undergone BM transplantation. CE–CT revealed a large solid mass of the ascending colon consistent with MS.

## Patient consent

Informed consent for publication of their case was obtained from the patient.
